# Impact of Ginger on Gut Microbiota Composition and Function in a *Bacteroides*-Dominant Enterotype

**DOI:** 10.4014/jmb.2503.03032

**Published:** 2025-05-26

**Authors:** Jinwoo Kim, Jina Ha, Seongok Kim, Gyungcheon Kim, Hakdong Shin

**Affiliations:** 1 Department of Food Science and Biotechnology, College of Life Science, Sejong University, Seoul 05006, Republic of Korea; 2 Carbohydrate Bioproduct Research Center, Sejong University, Seoul 05006, Republic of Korea

**Keywords:** Ginger, gut microbiota, enterotype, proteobacteria, dietary intervention

## Abstract

Ginger (*Zingiber officinale*) has been used worldwide for centuries, valued for both its culinary applications and potential therapeutic properties. Its bioactive compounds exhibit antioxidant, anti-inflammatory, and metabolic regulatory effects, providing physiological benefits to the human body. However, its influence on the gut microbiota remains poorly understood. In this study, we investigated the impact of ginger on gut microbiota using an *in vitro* fecal incubation model. To minimize interindividual variability, we classified participants into enterotypes based on gut microbial composition, focusing on the *Bacteroides*-dominant enterotype. While ginger treatment did not significantly affect microbial alpha diversity, it induced distinct shifts in bacterial structure, suggesting compositional changes in the microbiota. At the phylum level, taxonomic analysis revealed a lower relative abundance of Bacteroidota and a higher relative abundance of Proteobacteria in the ginger-treated group compared to the control. Consistently, genus-level analysis showed an increased relative abundance of *Acinetobacter* and *Enterobacteriaceae*, both belonging to Proteobacteria, in the ginger-treated group. Predicted functional pathway analysis further revealed that ginger treatment enriched pathways related to linoleic acid metabolism, beta-alanine metabolism, geraniol degradation, and tetracycline biosynthesis. These findings suggest that ginger modulates gut microbiota composition, particularly by increasing the abundance of Proteobacteria-associated genera. This enterotype-based study provides a structured framework for evaluating dietary effects and may support the development of personalized dietary strategies targeting gut microbiome modulation.

## Introduction

The gut microbiome is a diverse and complex community of microorganisms, including bacteria, archaea, viruses, and fungi, that reside in the human gastrointestinal tract [1, 2]. These microbes dynamically interact with the host, playing a fundamental role in digestion, metabolism, immune regulation, and neurological function [3, 4]. The composition of the gut microbiota is shaped by multiple factors, such as genetics, age, and lifestyle [5]. Among these, diet is one of the most significant determinants of microbial structure, function, and diversity [6]. Certain dietary components, such as fiber and polyphenols, are resistant to digestion in the upper gastrointestinal tract and reach the colon, where they serve as substrates for microbial metabolism [7]. This process selectively enhances the growth of specific microbes, thereby shaping microbiome diversity and influencing host health. Investigating these diet-microbiome interactions can provide valuable insights into their impact on health outcomes and support the development of functional foods.

Ginger (*Zingiber officinale*) has been used for medicinal purposes for over 5,000 years [8]. It contains various bioactive compounds, including polyphenols and terpenes, which exhibit antioxidant, anti-inflammatory, anticarcinogenic, and antihyperglycemic effects [9, 10]. Beyond these physiological effects, ginger may also influence gut microbiota through its undigested bioactive components [11, 12]. Certain compounds, such as ginger-derived polyphenols and fiber-associated metabolites, can resist digestion in the upper gastrointestinal tract and reach the colon, where they interact with gut microbes [13]. While these compounds may have prebiotic potential by serving as substrates for microbial metabolism, their specific effects on gut microbiota remain poorly understood. Further research is needed to elucidate ginger’s role in shaping microbial composition and function.

Several strategies have been employed to investigate the effects of dietary components on the human gut microbiota [14]. Human intervention studies provide the most relevant insights by capturing host-microbiota interactions under physiological conditions. However, these human studies are limited by high costs and ethical considerations. Additionally, the inability to fully control sample collection and study design makes it challenging to assess the specific effects of individual dietary components on gut microbiota [15]. Animal models offer a controlled environment for dietary interventions, but fundamental differences in gut physiology, immune function, and microbial composition limit their applicability to humans [16]. As an alternative, *in vitro* fecal incubation systems have been developed to overcome these limitations [17]. While these models do not replicate host physiology, they allow for reproducible and targeted analysis of microbial responses to specific dietary substrates under controlled conditions.

In this study, we examined the effects of ginger on gut microbiota using an *in vitro* fecal incubation model. To minimize interindividual variability, we categorized fecal samples into enterotypes based on their microbial composition. Through enterotype-specific analysis of microbial shifts and functional pathway predictions, we aimed to assess ginger’s potential role in modulating gut microbiota dynamics.

## Materials and Methods

### Study Population

This study enrolled 24 healthy Korean participants through voluntary participation. None of the participants had a history of gastrointestinal disorders or had taken antibiotics within one month prior to the experiment. Fresh fecal samples were collected using sterile cotton-tipped swabs and immediately transferred to an anaerobic chamber for *in vitro* fecal culturing. Detailed participant information is provided in Table S1.

### *In vitro* Fecal Incubation

Fecal samples were transferred into an anaerobic workstation (Coy Laboratory Products Inc., USA) filled with a gas mixture of 5% H_2_, 5% CO_2_, and 90% N_2_. Samples were homogenized in MiPro medium [17], and filtered through a sterile 985 μm nylon mesh. The homogenate was then inoculated into 96-deep well plates, with the fecal concentration adjusted to 2.0% (w/v). Commercial ginger powder was purchased from a local market in Seoul, Republic of Korea. The ginger concentrations were selected based on a human intake level (3 g/day) demonstrated to exert physiological effects [18]. The median daily stool weight of healthy adults (106 g/day) was used as a reference [19]. Considering both intake and stool, treatment concentrations of 0.2 mg/ml (equivalent to 1.06 g of ginger per 106 g of feces) and 1.0 mg/ml (equivalent to 5.30 g of ginger per 106 g of feces) were applied. Fecal suspensions without ginger treatment served as controls. The plates were incubated anaerobically at 37°C, and samples were collected at 0 and 24 h. Samples taken at 0 h were used for enterotype analysis, and those collected at 24 h were analyzed for compositional changes. Incubated fecal samples were immediately stored at −80°C for further analysis.

### 16S rRNA Amplicon Analysis

Total DNA was extracted from the cultured samples using the HTP 96 DNA Extraction kit (Qiagen, Germany) according to the manufacturer's protocol. PCR amplification was then performed following the EMP standard protocol, using 515F/806R primers to target the V4 hypervariable region of the 16S rRNA gene [20]. The resulting amplicons were pooled and sequenced on the Illumina MiSeq platform using paired-end sequencing (2 × 300 cycles). 16S rRNA sequence analysis was conducted using the QIIME2 software package (version 2020.6), and the analyses were performed at the Biopolymer Research Center for Advanced Materials (BRCAM, Sejong University, Seoul, Republic of Korea) [21]. DADA2 was used for trimming and denoising, generating amplicon sequence variants (ASVs), which were then aligned using MAFFT [22]. Taxonomic classification of ASVs was performed based on the SILVA 132 database [23, 24]. To minimize bias due to sequencing depth variation, rarefaction was applied to normalize read counts across samples.

### Stratification of Gut Microbiota Composition

The baseline gut microbiota composition was analyzed using DNA extracted from fecal culture samples collected at 0 h. Clustering was performed based on genus-level relative abundances using the Jensen-Shannon distance (JSD) and the Partitioning Around Medoids (PAM) clustering algorithm [25]. The optimal number of clusters was determined by maximizing the Calinski-Harabasz (CH) index, and cluster validation was assessed using the silhouette coefficient. Clustering results were visualized using Principal Coordinates Analysis (PCoA).

### Prediction of Functional Metagenomic Profiles

Functional metagenomic profiling was performed using 16S rRNA gene sequences with Phylogenetic Investigation of Communities by Reconstruction of Unobserved States 2 (PICRUSt2, version 2.3.0) [26]. Kyoto Encyclopedia of Genes and Genomes (KEGG) ortholog (KO) abundances were inferred to predict potential enzymatic activities. Functional differences between groups were assessed using linear discriminant analysis effect size (LEfSe), with an LDA score threshold of >3.0 and an adjusted *p*-value < 0.05 [27]. The Benjamini-Hochberg method was applied to adjust *p*-values and control the false discovery rate.

### Quantification and Statistical Analysis

Statistical comparisons across groups were conducted using the Kruskal-Wallis test, followed by pairwise comparisons with the Mann-Whitney U test. Diversity and multivariate analyses were performed using MicrobiomeAnalyst (version 2.0) [28]. Group differences in PCoA plots were evaluated using permutational multivariate analysis of variance (PERMANOVA). To control the false discovery rate, all *p*-values were adjusted using the Benjamini-Hochberg method.

### Data Availability

Amplicon sequence data and corresponding metadata are publicly accessible through the EMP data portal on Qiita (https://qiita.ucsd.edu; study ID: 15896).

### Institutional Review Board Statement

This study was approved by the Institutional Review Board (IRB) of Sejong University (IRB no. SJU-BR-E-2020-025). Participation was voluntary, and written informed consent was obtained from all participants.

## Results

### Enterotype Classification

**Fig. 1:**
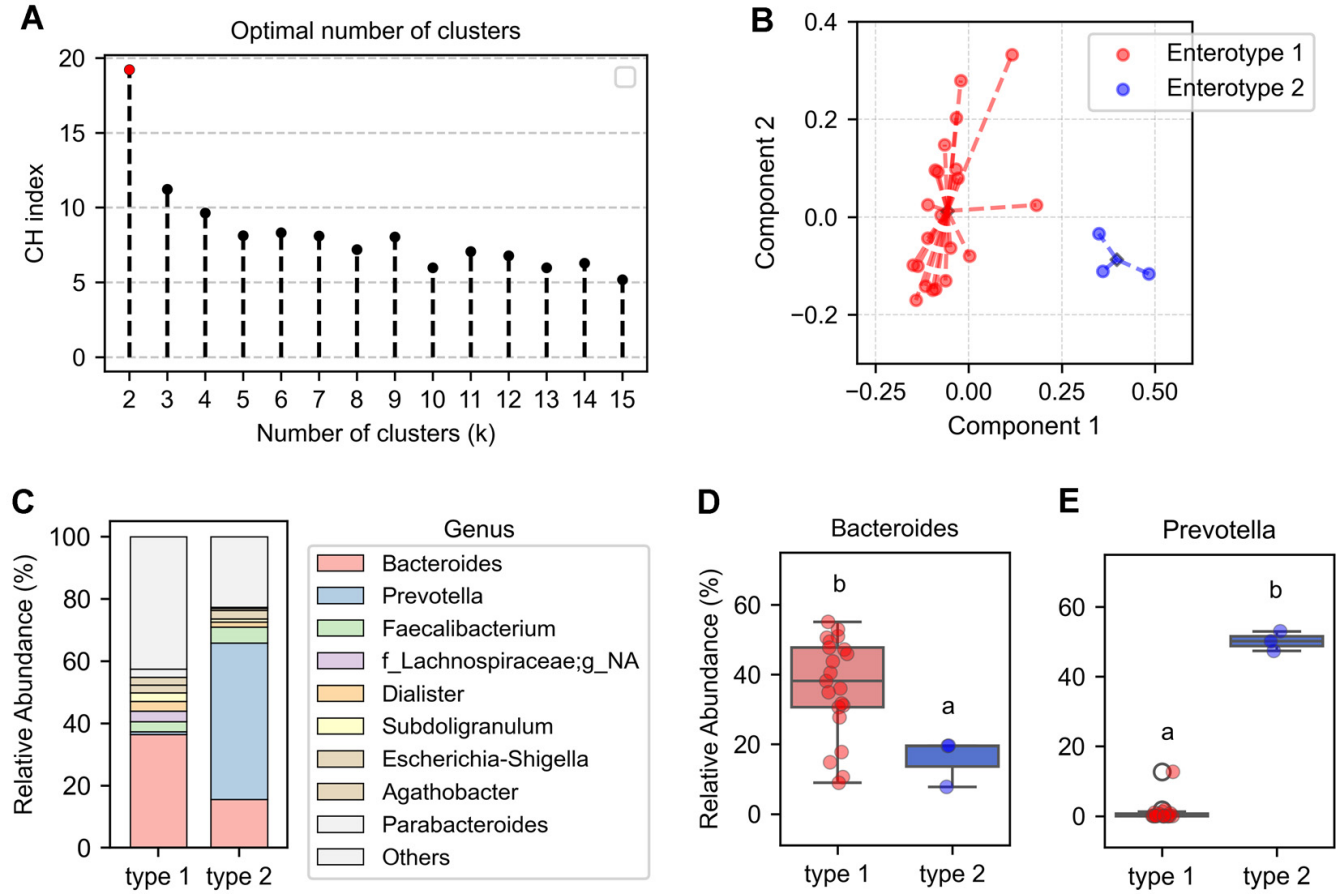
Enterotype classification of gut microbiota. (**A**) The optimal number of clusters was determined using the Calinski-Harabasz (CH) index. CH index values were calculated for cluster numbers (k) ranging from 2 to 15, with the highest value indicating the optimal clustering, marked with a red dot. (**B**) Principal coordinates analysis was performed based on relative genus-level abundances using the Jensen–Shannon divergence distance matrix and the partitioning around medoids clustering algorithm. Each circle represents a sample, with colors distinguishing the two enterotypes. Dashed lines indicate the distance of each sample from its respective cluster centroid (diamond). (**C**) Relative abundance of dominant bacterial genera in each enterotype. g_NA denotes unassigned genera at the genus level. (**D-E**) The relative abundance of *Bacteroides* (**D**) and *Prevotella* (**E**) was compared between the two enterotypes, with significant differences indicated by different letters (a, b).

To minimize the effects of individual variability, we performed enterotype analysis on gut microbiota from 24 fecal samples collected from Korean participants. Enterotypes were classified based on gut microbial community composition. Clustering analysis was conducted using the Jensen-Shannon distance (JSD) at the genus level and the Partitioning Around Medoids (PAM) algorithm. The optimal number of clusters was determined using the Calinski-Harabasz (CH) index (Fig. 1A).

Clustering analysis revealed two distinct enterotypes (Fig. 1B). To characterize these enterotypes, we examined the relative abundance of genera in each group (Fig. 1C). Enterotype 1 was predominantly characterized by *Bacteroides* (Fig. 1C and 1D), whereas Enterotype 2 exhibited a higher relative abundance of *Prevotella* (Figs. 1C and 1E). As enterotypes are distinguished by their dominant genera [25], we further analyzed individual sample compositions. Among the 24 samples, 19 were classified as *Bacteroides*-dominant, 3 as *Prevotella*-dominant, and the remaining 2 were dominated by *Megamonas* and *Eubacterium*, respectively (Table S1).

Due to the small sample size of non-*Bacteroides*-dominant groups (n ≤ 3 per group), they were excluded from further analysis. Subsequent analyses focused exclusively on the *Bacteroides*-dominant group, as all individuals in this group were classified as Enterotype 1.

### Impact of Ginger on Alpha Diversity in *Bacteroides*-Dominant Fecal Microbiota

**Fig. 2:**
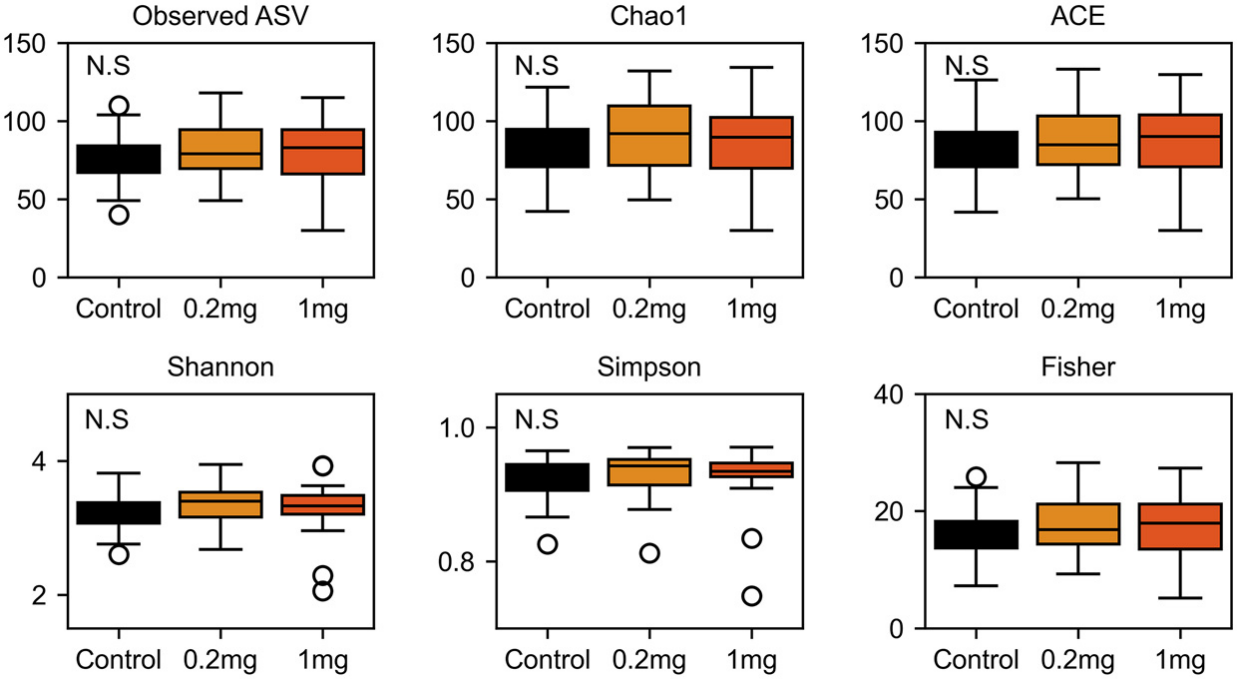
Alpha diversity in response to ginger treatments. Alpha diversity was assessed using the number of observed amplicon sequence variants (ASVs), Chao1, and ACE indices for species richness, as well as Shannon, Simpson, and Fisher indices for microbial diversity and evenness. Statistically non-significant differences are indicated as "N.S".

We examined the effect of ginger on the gut microbiota using an *in vitro* fecal incubation system [17]. Fecal samples were incubated under anaerobic conditions in a homogenization medium containing either 0.2 mg/ml or 1.0 mg/ml of ginger. A total of 1,332,211 paired-end sequences (Phred score >Q20) were obtained across all groups, with an average of 18,503 sequences per sample (Table S2). Analysis focused on the *Bacteroides*-dominant subgroup, which included 19 samples. Alpha diversity was assessed using observed ASVs, Chao1, ACE, Shannon, Simpson, and Fisher indices (Fig. 2). No significant differences were observed in any of the indices among the control, 0.2 mg, and 1.0 mg ginger-treated groups.

### Changes in Fecal Microbial Communities Induced by Ginger Treatment

**Fig. 3:**
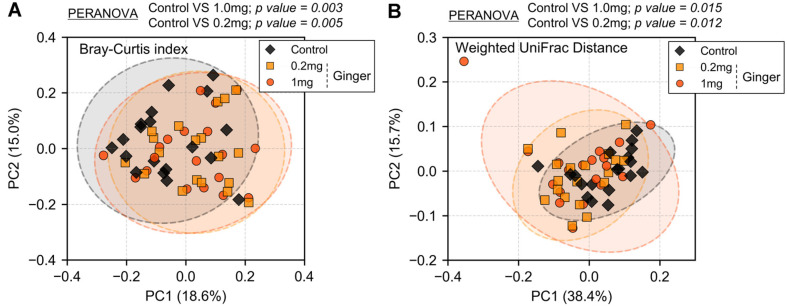
Ginger-induced shifts in microbial community composition. Beta diversity was analyzed using principal coordinates analysis based on the Bray-Curtis index (**A**) and Weighted UniFrac distance (**B**). Each point represents a sample, with the control (black diamonds), 0.2 mg ginger (orange squares), and 1 mg ginger (red circles). Ellipses indicate 95% confidence intervals. Statistical significance was assessed using permutational multivariate analysis of variance (PERMANOVA), and *p*-values were adjusted for multiple comparisons using the Benjamini-Hochberg method.

Beta diversity analysis was performed to evaluate structural shifts in the fecal microbiota following ginger treatment. Principal coordinates analysis (PCoA) plots, based on the Bray-Curtis index (Fig. 3A) and weighted UniFrac distances (Fig. 3B), revealed distinct clustering between the control and ginger-treated groups.

Pairwise permutational multivariate analysis of variance (PERMANOVA) confirmed significant alterations in microbial community composition. According to the Bray-Curtis index, both the 0.2 mg and 1.0 mg ginger-treated groups differed significantly from the control group (PERMANOVA, *p* = 0.005 and *p* = 0.003, respectively). Similarly, the weighted UniFrac analysis indicated significant differences between the control and ginger-treated groups (PERMANOVA, *p* = 0.012 for 0.2 mg and *p* = 0.015 for 1.0 mg), while no significant differences were observed between the two ginger-treated groups. These findings suggest that ginger treatment can modulate overall gut microbiota composition in *Bacteroides*-dominant samples.

### Key Fecal Microbial Taxa Altered by Ginger Treatment

**Fig. 4:**
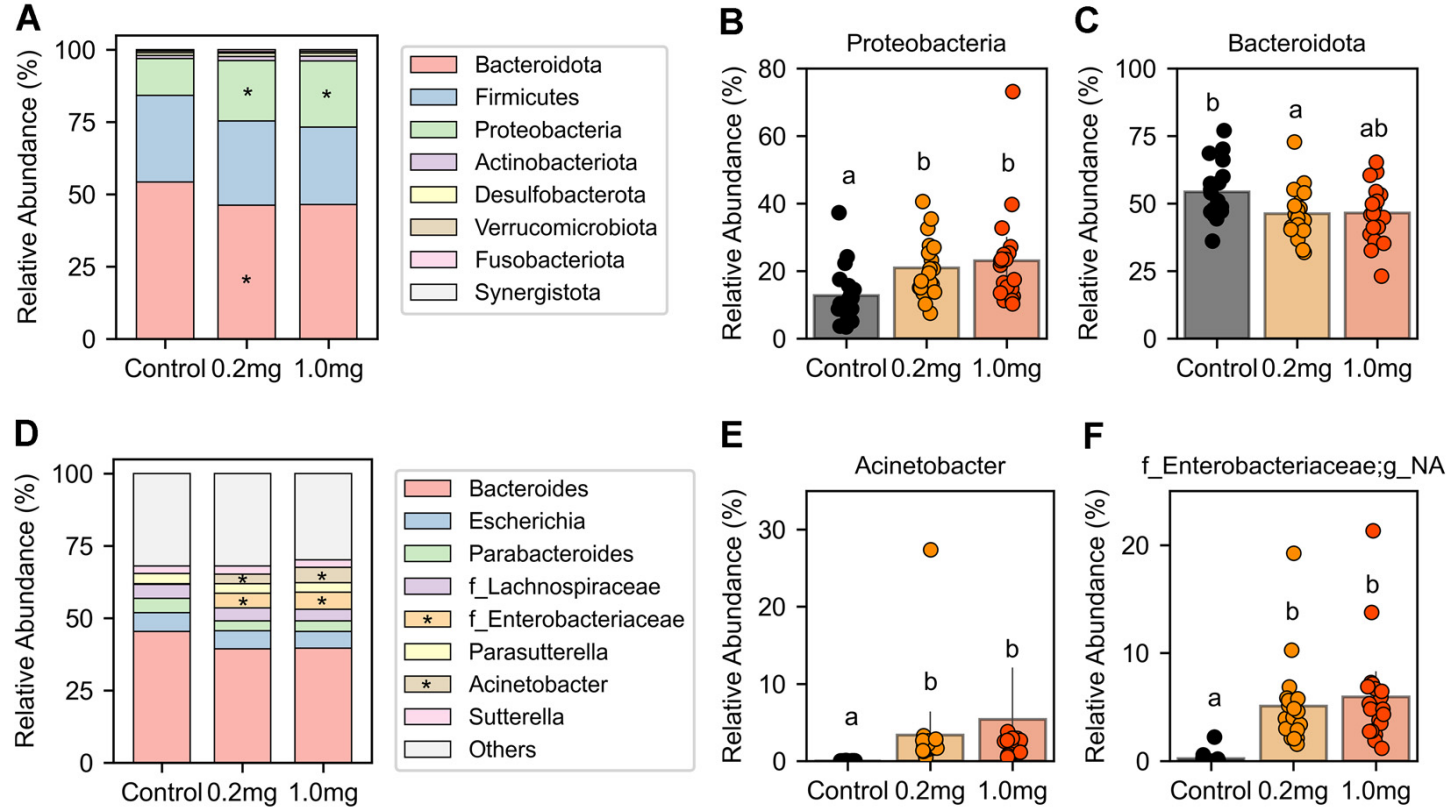
Impact of ginger on gut microbial composition. (**A**) The effect of ginger treatment on gut microbial composition was assessed by comparing the relative abundance of bacterial phyla across the control, 0.2 mg, and 1 mg ginger-treated groups. (**B-C**) Proteobacteria (**B**) and Bacteroidota (**C**) were identified as differentially abundant through Linear Discriminant Analysis Effect Size (LEfSe) and compared between groups. (**D**) The relative abundance of the top eight bacterial genera was analyzed, with genera averaging less than 1% across the three groups grouped as "Others". (**E-F**) The relative abundance of *Acinetobacter* (**E**) and an unassigned genus (g_NA) within the family Enterobacteriaceae (**F**) was compared. Asterisks (*) indicate phyla or genera with significant differences between groups. Different letters (a, b) denote statistically significant differences.

We analyzed bacterial composition at the phylum level to evaluate the impact of ginger on microbial community structure. A total of eight phyla were identified: Bacteroidota, Firmicutes, Proteobacteria, Actinobacteriota, Desulfobacterota, Verrucomicrobiota, Fusobacteriota, and Synergistota (Fig. 4A).

To determine which phyla exhibited significant differences between groups, we performed linear discriminant analysis effect size (LEfSe) analysis, applying an LDA score threshold of 3.0 and an adjusted *p*-value cutoff of 0.05. This analysis revealed significant changes in the relative abundances of Bacteroidota and Proteobacteria following ginger supplementation (Fig. 4B and 4C). Specifically, the relative abundance of Bacteroidota was reduced in both the 0.2 mg (*p* = 0.046) and 1.0 mg (*p* = 0.081) ginger-treated groups compared to the control. In contrast, Proteobacteria showed a significant increase in both the 0.2 mg (*p* = 0.003) and 1.0 mg (*p* = 0.005) groups, with no significant difference between the two ginger-treated groups (*p* = 0.96).

To further investigate taxonomic differences, we performed a genus-level analysis (Fig. 4D). LEfSe analysis identified *Acinetobacter* and an unassigned genus within the family *Enterobacteriaceae* as significantly enriched in the ginger-treated groups (*p* < 0.001). Both genera belong to the phylum Proteobacteria and exhibited a higher relative abundance in the ginger-treated groups than in the control (Fig. 4E and 4F). No significant difference was observed between the two ginger-treated groups for either *Acinetobacter* (*p* = 0.321) or the unassigned genus within the family *Enterobacteriaceae* (*p* = 0.397).

### Predicted Functional Metabolic Pathway

To predict functional pathways associated with ginger treatment in the gut microbiome, we performed Phylogenetic Investigation of Communities by Reconstruction of Unobserved States 2 (PICRUSt2) analysis, based on Kyoto Encyclopedia of Genes and Genomes (KEGG) pathways. LEfSe analysis (*p* < 0.05, LDA score > 3.0) was applied to identify differentially enriched pathways between the control and ginger-treated groups.

Ginger treatment resulted in significant alterations in multiple metabolic pathways (Table 1). Both the 0.2 mg and 1.0 mg ginger-treated groups exhibited significant enrichment in pathways related to lipid metabolism (linoleic acid metabolism), amino acid metabolism (beta-alanine metabolism), and terpenoid and polyketide metabolism (geraniol degradation and tetracycline biosynthesis). In contrast, the control group demonstrated significant enrichment in pathways associated with xenobiotic biodegradation (naphthalene degradation).

## Discussion

We examined the impact of ginger on the gut microbiota using an *in vitro* fecal incubation model. Although this model does not fully replicate host physiological responses and intestinal absorption, it offers a controlled and reproducible system for screening microbial responses to dietary substrates under standardized conditions [29]. To improve the accuracy of our analysis using this model, we applied an enterotype-based approach by classifying the gut microbiota of 24 healthy Korean participants. This approach provides a structured framework for analyzing gut microbiota response patterns and may support the development of personalized dietary strategies targeting gut microbiome modulation.

Ginger treatment did not significantly impact microbial alpha diversity (Fig. 2). However, beta diversity analysis revealed significant compositional differences between the control and ginger-treated groups (Fig. 3). LEfSe analysis further identified differentially abundant taxa between the groups. At the phylum level, Bacteroidota exhibited a lower relative abundance in the ginger-treated groups, whereas Proteobacteria showed an increased relative abundance following ginger treatment (Fig. 4A-4C). Genus-level analysis revealed that *Acinetobacter* and an unassigned genus within *Enterobacteriaceae*, both belonging to Proteobacteria, were more abundant in the ginger-treated groups (Fig. 4D-4F).

Interindividual variability in gut microbiota poses a challenge in analyzing diet-microbiome interactions [30]. To address this issue, an enterotype-based approach was developed to classify individuals based on microbiota composition and predominant bacterial genera [25]. This classification provides a structured framework for assessing dietary responses within similar gut microbiota profiles, thereby reducing interindividual variability. A previous study classified the human gut microbiome into three main enterotypes: *Bacteroides*-dominant, *Prevotella*-dominant, and *Ruminococcus*-dominant, each characterized by distinct microbial compositions and metabolic functions [25]. These enterotype-specific differences influence microbial interactions with dietary components, leading to distinct metabolic responses [31]. In this study, we applied an enterotype-based approach to examine the effects of ginger on gut microbiota composition and dynamics. Among the 24 participants, 19 were classified as *Bacteroides*-dominant, while the remaining samples included *Prevotella*-dominant (*n* = 3), *Megamonas*-dominant (*n* = 1), and Eubacterium-dominant (*n* = 1) (Table S1). Due to the small number of non-*Bacteroides*-dominant samples, analyses were restricted to the *Bacteroides*-dominant group. This study provides insights into microbiota changes induced by ginger within the *Bacteroides*-dominant enterotype. While this approach helped minimize interindividual variability, the focus on a single enterotype limits the generalizability of the findings. A larger and more diverse cohort is needed to validate these observations across different enterotype groups.

Taxonomic analysis revealed that ginger treatment at concentrations of 0.2 mg/ml (equivalent to 1.06 g of ginger per 106 g of feces) and 1.0 mg/ml (equivalent to 5.30 g of ginger per 106 g of feces) increased the relative abundance of Proteobacteria in the *Bacteroides*-dominant enterotype. This observation aligns with previous studies suggesting that ginger may influence gut microbial composition by promoting the growth of Proteobacteria. A human clinical trial involving 123 healthy adults reported a significant increase in Proteobacteria abundance following short-term fresh ginger juice consumption (30 g/day, equivalent to 500 mg/kg/day for a 60 kg individual, over 7 days) [32]. Additionally, an animal study demonstrated that ginger powder supplementation (500 mg/kg/day for 16 weeks) led to an increase in Proteobacteria levels [33]. Our model did not mimic digestive processes, suggesting that the applied ginger concentrations in this study require further investigation. However, previous studies have reported similar effects of ginger on Proteobacteria abundance. These *in vivo* studies support the association between ginger consumption and Proteobacteria enrichment, reinforcing the relevance of our *in vitro* findings.

Ginger contains various bioactive compounds with antioxidant, anti-inflammatory, and antimicrobial properties [13]. Among these, gingerols are the primary bioactive components in fresh ginger. As polyphenolic compounds, they contribute to free radical scavenging, oxidative stress reduction, and inflammation modulation [34]. In addition to these physiological effects, gingerols have been shown to influence microbial growth. Studies have demonstrated that they inhibit biofilm formation, suppress quorum sensing, and disrupt membrane integrity in pathogens such as *Pseudomonas aeruginosa* and *Staphylococcus aureus* [35, 36]. These findings suggest that gingerols may play a role in shaping microbial composition, but their specific impact on Proteobacteria remains unclear. Further research is needed to determine whether Proteobacteria can metabolize ginger-derived compounds or if ginger treatment affects their growth.

To predict metabolic pathways, we performed PICRUSt2 analysis. Our results indicated significant enrichment of pathways related to xenobiotic biodegradation, terpenoid metabolism, and amino acid metabolism in the ginger-treated group (Table 1). However, the precise effects of ginger-derived compounds on microbial metabolic activity remain unclear. This study relied on 16S rRNA gene sequencing, which provides taxonomic information but limited functional resolution. To address this limitation, complementary approaches such as shotgun metagenomics and metabolomics are necessary. Shotgun metagenomic sequencing enables genome-wide characterization of microbial genes and metabolic pathways, offering deeper insights into functional potential beyond taxonomic composition [37]. Metabolomics further enhances functional interpretation by profiling metabolites produced through microbial activity [38]. Applying such multi-omics strategies could provide a more comprehensive understanding of how ginger modulates the composition and functional dynamics of the gut microbiota.

Our study has several limitations. First, the sample size was relatively small, and the analysis was restricted to the *Bacteroides*-dominant enterotype. Considering other enterotypes, including the *Prevotella*-dominant and *Ruminococcus*-dominant types [25], a more diverse population should be analyzed to gain a comprehensive understanding of ginger's impact on the gut microbiota. Additionally, the *in vitro* fecal incubation model used in this study did not incorporate digestive processes such as enzymatic breakdown, absorption, and metabolism that occur before ginger reaches the colon. As a result, the applied concentrations may not accurately reflect the actual amount of ginger reaching the colon, potentially leading to exaggerated effects. However, intake levels of ginger associated with physiological effects in humans vary widely, typically ranging from 1 g to 30 g per day [18, 32, 39]. The microbial changes observed were consistent with findings from *in vivo* studies, supporting the validity of our approach [32]. To improve the physiological relevance of *in vitro* models, future studies should include simulated digestion steps or test a broader range of ginger concentrations.

Our findings suggest that ginger modulates gut microbiota composition. Although this study focused solely on the *Bacteroides*-dominant enterotype, an enterotype-based approach offers a structured framework for evaluating dietary effects on microbiota composition. This strategy not only helps mitigate interindividual variability but also supports the development of personalized dietary interventions to enhance gut health.

## Supplemental Materials

Supplementary data for this paper are available on-line only at http://jmb.or.kr.



## Figures and Tables

**Table 1:** Impact of ginger on predicted functional pathways of gut microbiota.

Comparison	Pathway	Level 3	Enriched Group	LDA	P-value
Control vs Ginger 0.2 mg	Xenobiotics biodegradation and metabolism	Naphthalene degradation	Control	3.048	<0.001
	Metabolism of terpenoids and polyketides	Tetracycline biosynthesis	Ginger	3.237	<0.001
	Lipid metabolism	Linoleic acid metabolism	Ginger	3.231	<0.001
	Metabolism of terpenoids and polyketides	Geraniol degradation	Ginger	3.079	<0.001
	Metabolism of other amino acids	Beta-Alanine metabolism	Ginger	3.397	<0.001
Control vs Ginger 1 mg	Xenobiotics biodegradation and metabolism	Naphthalene degradation	Control	3.066	<0.001
	Metabolism of terpenoids and polyketides	Tetracycline biosynthesis	Ginger	3.231	<0.001
	Lipid metabolism	Linoleic acid metabolism	Ginger	3.245	<0.001
	Metabolism of terpenoids and polyketides	Geraniol degradation	Ginger	3.178	<0.001
	Metabolism of other amino acids	Beta-Alanine metabolism	Ginger	3.412	<0.001
