# Supplementary material for: Impact of Ginger on Gut Microbiota Composition and Function in a Bacteroides-Dominant Enterotype
Source: J Microbiol Biotechnol. 2025 May 26;35:e2503032. doi: 10.4014/jmb.2503.03032 (PMC12149393; doi:10.4014/jmb.2503.03032)
Supplement: Supplementary file 1 [file jmb-35-e2503032-supple.pdf]

J. Microbiol. Biotechnol. 2025. 35: e2503032
https://doi.org/10.4014/jmb.2503.03032

Supplementary Tables

Impact of Ginger on Gut Microbiota Composition and Function in a *Bacteroides*-Dominant Enterotype

Jinwoo Kim[1,2†], Jina Ha[1†], Seongok Kim[1,2], Gyungcheon Kim[1], and Hakdong Shin[1,2*]

[1]Department of Food Science and Biotechnology, College of Life Science, Sejong University, Seoul 05006, Republic of Korea

[2]Carbohydrate Bioproduct Research Center, Sejong University, Seoul 05006, Republic of Korea

†These authors contributed equally to this work.

*Correspondence to: hshin@sejong.ac.kr (H.S.)

Table S1. Classification of gut microbiota and dominant genera

SampleID	Type[a]	Dominant genus[b]	P/B[c]
Sub.001	1	*Bacteroides*	0.001
Sub.002	1	*Bacteroides*	<0.001
Sub.003	1	*Bacteroides*	<0.001
Sub.004	1	*Bacteroides*	<0.001
Sub.006	1	*Bacteroides*	<0.001
Sub.007	1	*Bacteroides*	<0.001
Sub.008	1	*Bacteroides*	0.002
Sub.009	1	*Bacteroides*	<0.001
Sub.010	1	*Bacteroides*	<0.001
Sub.011	1	*Bacteroides*	<0.001
Sub.012	1	*Bacteroides*	<0.001
Sub.013	1	*Bacteroides*	0.03
Sub.014	1	*Bacteroides*	0.02
Sub.018	1	*Bacteroides*	<0.001
Sub.019	1	*Bacteroides*	0.286
Sub.020	1	*Bacteroides*	0.015
Sub.025	1	*Bacteroides*	0.002
Sub.027	1	*Bacteroides*	0.021
Sub.030	1	*Bacteroides*	<0.001
Sub.015	1	*Megamonas*	<0.001
Sub.017	1	*Eubacterium*	0.168
Sub.005	2	*Prevotella*	0.709
Sub.016	2	*Prevotella*	0.872
Sub.022	2	*Prevotella*	0.719

[a]Samples were classified into two groups based on genus-level relative abundance using JSD distance and PAM clustering. [b]The most abundant genus in each sample's gut composition. [c]Proportion of *Prevotella* relative to the sum of *Bacteroides* and *Prevotella*.

Table S2. Sequence information

Group	Control	Ginger 0.2mg	Ginger 1mg
Number of samples	24	24	24
Total number of sequences (seqs)	408,941	468,984	454,286
Mean number of sequences ± SD[a]	17,039 ± 9,490	19,541 ± 11,842	18,929 ± 12,358
Number of observed ASV[b]	840	945	909

[a]SD, Standard Deviation; [b]ASV, Amplicon Sequence Vari
